# Case Report: First In-Human Report of Cure of Refractory *Mycoplasma genitalium* Infection With Omadacycline

**DOI:** 10.1093/ofid/ofag030

**Published:** 2026-03-30

**Authors:** Nisha M Patel

**Affiliations:** Department of Medicine, Division of Infectious Diseases, NYU Grossman School of Medicine, NewYork, New York, USA

**Keywords:** multidrug-resistant *Mycoplasma genitalium*, omadacycline, prostatitis, urethritis, urogenital infection

## Abstract

*Mycoplasma genitalium* (MG) is increasingly recognized as a sexually transmitted cause of nongonococcal urogenital infections. MG demonstrates high rates of antimicrobial resistance, and there are few available treatment options for infections caused by multidrug-resistant organisms. There is a paucity of guidance on the management of MG infections in which initial therapy fails, and additional globally accessible treatments are needed to address this rising clinical challenge. This report describes the first in-human case of refractory MG infection that was cured after treatment with omadacycline.

## CASE REPORT

An immunocompetent man in his 20s, not living with human immunodeficiency virus type 1 (HIV-1) and on medication for HIV prevention, experienced urethritis symptoms in August 2024, described as penile discomfort and white urethral discharge. As a man who has sex with men, he reported sexual contact in the time before onset of his symptoms but no known specific exposure. At his primary care clinic, he tested negative for gonorrhea and chlamydia and was given empiric treatment with intramuscular ceftriaxone, oral azithromycin, and oral amoxicillin-clavulanate. In September 2024, about week 5 since symptom onset, the patient tested positive for *Mycoplasma genitalium* (MG) with urine polymerase chain reaction (PCR) testing. He was treated with sequential doxycycline, 100 mg twice daily for 7 days, followed by moxifloxacin, 400 mg/d for 7 days.

The patient experienced some improvement while taking doxycycline but had recurrence of the same symptoms with moxifloxacin. Urinalysis and urine culture performed at week 9 of symptoms revealed no evidence of other bacterial infection. At week 10, he saw a urologist, who observed a boggy prostate on examination and recommended empiric treatment for bacterial prostatitis with trimethoprim-sulfamethoxazole for 14 days. While the patient’s symptoms and prostate examination findings were initially mildly improved, his symptoms of penile pain and white urethral discharge recurred with increased intensity. At week 12 of symptoms, he was again prescribed doxycycline, 100 mg twice daily for 8 days, and trimethoprim-sulfamethoxazole, for 8 days.

Given his ongoing symptoms, the patient was advised to seek a second opinion, and he presented to the infectious diseases clinic in December 2024. By that time, his urethritis symptoms had been ongoing for 20 weeks. He was highly symptomatic, with thick penile discharge, pain at the tip of the penis, and dysuria. He reported no sexual intercourse in the preceding 4 months, since the urethritis symptoms had initially developed in August 2024. After his evaluation in the infectious diseases clinic, his symptoms were deemed likely due to persistent MG infection, with the alterative consideration of prostatitis due to typical bacteria, such as enteric or genitourinary flora. He was treated with minocycline, 100 mg twice daily for 14 days directed against urogenital MG infection, and simultaneously started again on an empiric course of trimethoprim-sulfamethoxazole for 6 weeks. He reported an improvement in symptoms while taking minocycline, characterized by diminished urethral discharge and penile pain, followed by recurrence when minocycline was stopped. Results of urine MG PCR, performed 13 days after completion of minocycline treatment, remained positive, and the patient’s symptoms of urethral discharge, penile pain, and dysuria persisted. Resistance to macrolides was presumed based on the known prevalence of macrolide resistance; testing for such resistance was not performed due to it unclear clinical benefit.

Due to the patient's persistent urethritis symptoms and positive urine MG PCR, salvage therapy was initiated around week 23 of symptoms with sequential minocycline, 100 mg twice daily for 7 days, followed by tinidazole, 2 g/d for 14 days. Again, the patient experienced improvement with minocycline but significant worsening with tinidazole. At this point, multiple lines of therapy had failed, and the patient remained symptomatic with urethritis, though the intensity of his symptoms fluctuated. He reported feeling hopeless, anxious, and fearful. He consistently reported worsening symptoms after masturbation, which was postulated to be caused by a reservoir of infection in the prostate. Magnetic resonance imaging of the prostate revealed no abscess but did show inflammation suggestive of prostatitis.

Minocycline treatment was resumed, with the intention of providing symptom relief while waiting for insurance authorization and copayment assistance for omadacycline. The patient took minocycline, 100 mg twice daily for 15 days, and then by week 30 of symptoms he switched to omadacycline, 300 mg/d for 14 days, to address urethritis and prostatitis due to MG. He had mild improvement of his symptoms with minocycline, and by day 9 of omadacycline his symptoms had nearly resolved. However, he experienced a transient increase in urethral discomfort and dysuria around day 10 of omadacycline, followed by minimal symptoms after completion of therapy. Fourteen days after completion of omadacycline treatment, his symptoms remained minimal, and urine MG PCR results were negative. Results of postmasturbation urinalysis were also normal. At this point, it was presumed that the patient was asymptomatic from MG infection, but lingering urethral discomfort was likely due to postinfectious inflammatory changes. Naproxen was prescribed to reduce residual inflammation of the urethra and prostate. Approximately 68 days after completion of omadacycline treatment and 43 weeks since symptoms started, results of repeated urine MG PCR remained negative. The patient’s symptoms, diagnostic evaluations, treatment, and symptomatic response are detailed in Table 1.

**Table 1.** Summary of Symptoms, Diagnostic Evaluation, Treatment, and Response

Time Since Onset of Symptoms, wk	Symptoms	Examination/Diagnostic Testing	Treatment	Response
1	Urethral discharge and pain in penis	Urine GC/CT negative	Ceftriaxone ×1 dose	Improved, then recurred
3	Urethral discharge	3-Site GC/CT negative; RPR negative	Azithromycin ×7 d; amoxicillin-clavulanate ×7 d	Persistent
5	Urethral discharge	MG PCR positive	Doxycycline ×7 d; moxifloxacin ×7 d	Improved with doxycycline, recurred with moxifloxacin; possible adverse effect from moxifloxacin
9	Urethral discharge and pain in penis	UA/UCx negative	Doxycycline ×7 d	Mildly persistent
10	Urethral discharge and pain in penis	Boggy prostate	TMP-SMX ×14 d	Nearly resolved
12	Urethral discharge and pain in penis	MG PCR positive; HIV, RPR, and pharyngeal GC/CT negative; *Trichomonas* negative	Doxycycline ×8 d; TMP-SMX ×8 d	Improved on doxycycline, then recurred
20 (presentation to ID clinic)	Urethral discharge, pain in penis, and dysuria	UA/UCx negative	Minocycline ×14 d; TMP-SMX ×6 wk	Improved on minocycline, then recurred
23	Urethral discharge, pain in penis, and dysuria	MG PCR positive; urine GC/CT negative; HIV negative	Minocycline ×7 d, then tinidazole ×14 d	Improved with minocycline, much worse recurrence with tinidazole, then mild
29	Mild urethral discharge and pain in penis	Prostate MR imaging: no abscess but findings suggestive of prostatitis	Minocycline ×15 d, then omadacycline ×14 d	Improved with minocycline, near resolution by d 9 of omadacycline, then recurrent discomfort in urethra and dysuria; minimal symptoms after completion of omadacycline
35	Minimal/none	MG PCR negative; postmasturbation UA negative	Naproxen ×7 d	…
43	Minimal/none	MG PCR negative	…	…

Abbreviations: GC/CT, gonorrhea/chlamydia; HIV, human immunodeficiency virus; ID, infectious diseases; MG, *mycoplasma genitalium*; MR, magnetic resonance; PCR, polymerase chain reaction; RPR, rapid plasma reagin; TMP/SMX, trimethoprim-sulfamethoxazole; UA, urinalysis; UCx, urine culture.

The patient provided written consent. This report does not include elements that require approval from the local institutional review board

## DISCUSSION

This is the first in-human report of omadacycline used to cure treatment-refractory and presumed multidrug-resistant (MDR) MG infection. After 33 weeks of symptoms and multiple failed lines of therapy, clinical cure was achieved after a 14-day course of omadacycline.

The rise of macrolide- and fluoroquinolone-resistant MG infections is increasingly recognized and represents a significant clinical challenge [1]. Numerous factors contribute to the difficulty with diagnosis and management. First, MG is a slow-growing organism that can be cultured only in research laboratories [2], and susceptibility testing is based on growth from culture. There is a lack of widely available PCR-based resistance gene testing. At the time of writing, the only commercially available drug-resistance testing for MG is available through ARUP Laboratories (https://ltd.aruplab.com/Tests/Pub/3006344) and is limited to testing for macrolide resistance. Such a test has low clinical value, given the already well-established high rates of macrolide resistance [3], especially among men who have sex with men [1].

Second, there is a lack of consensus guidance on treatment options in cases in which multiple therapies have failed. The 2021 sexually transmitted infection treatment guidelines from the US Centers for Disease Control and Prevention recommend expert consultation after failure of either sequential doxycycline and moxifloxacin or doxycycline and azithromycin [2]. Of course, not all providers necessarily have access to expert clinicians who are experienced with treating MDR urogenital infections. European guidelines suggest addition of pristinamycin as a third-line therapy option [4], but this medication is difficult to access outside Europe due to manufacturing and export issues [1]. A case series demonstrated success with a novel salvage therapy that entails a combination of methenamine, metronidazole, minocycline and pristinamycin [5], but this treatment strategy is limited to European and other countries that can access pristinamycin. Other medications that have demonstrated in vitro activity against MG, such as lefamulin [6] or sitafloxacin [1] are also not available in the United States. As such, there is a high need for treatment options that are not only efficacious but also globally accessible.

In this case, minocycline was initially used after failure of sequential doxycycline and moxifloxacin based on case reports of treatment success [7] and published efficacy data [8, 9] showing a 66.7%–71% cure rate with 14 days of minocycline. After failure of this therapy, sequential minocycline followed by tinidazole was tried, based on review of in vitro susceptibility testing [10] and anecdotal experience shared by clinician experts. After this patient's treatment period, a study was published on the successful use of combination therapy with metronidazole and minocycline [1], which may have been a suitable alternative treatment option. After the patient's symptoms significantly worsened with tinidazole treatment, it was clear that previous use of tetracyclines (doxycycline and minocycline) had provided incomplete but effective symptomatic relief. At this point, omadacycline was pursued as a fourth-line salvage treatment option.

Omadacycline is a tetracycline derivative that was approved by the US Food and Drug Administration in 2018 for treatment of acute bacterial skin and skin structure infections as well as community-acquired bacterial pneumonia [11]. It has broad in vitro activity, including against atypical bacteria and MDR organisms. Omadacycline also demonstrates high in vitro activity against MG, with significantly lower minimum inhibitory concentrations than other tetracyclines [12].

The patient was able to achieve clinical cure after a 15-day lead-in period with minocycline followed by 14 days of omadacycline. The minocycline was prescribed to provide symptom relief and theoretically may have reduced the bacterial load before initiation of omadacycline. It is notable that the patient experienced transient worsening of his symptoms while taking omadacycline; his eventual clinical cure demonstrates that progression of symptoms may not be linear and that transient worsening of symptoms may not indicate treatment failure. The progression of his infection from urethritis to prostatitis raised questions regarding the appropriate duration of therapy; while treatment durations for bacterial prostatitis are not well defined, 14 days of therapy may be sufficient [13]. This patient's treatment “failure” with initial treatment regimens may have been partly due to insufficient duration of therapy for a prostatic infection.

It is significant to note that the patient was able to obtain the full supply of omadacycline at no cost after insurance approval and financial assistance from the manufacturer, Paratek Pharmaceuticals. However, such financial assistance programs may not be available to all patients, and high cost may remain a significant barrier to use.

This case demonstrates that omadacycline may be a promising treatment option, as monotherapy or as a part of combination or sequential therapy for MDR MG infections. In the United States, there are few available treatment options after failure of sequential or combination treatment with doxycycline, moxifloxacin, azithromycin, minocycline, and nitroimidazoles (tinidazole and metronidazole). While it is important to acknowledge that broad conclusions cannot be based on a single case alone, this case demonstrates that further investigation is needed to explore the potential role of omadacycline in the treatment of MG infections.
